# Prevalence of Anemia and Associated Factors Among Undergraduate Students at SIMAD University, Mogadishu, Somalia

**DOI:** 10.1155/ah/6398491

**Published:** 2025-10-04

**Authors:** Fardowsa Mohamed Yusuf, Leena Babiker Mirghani, Abdirasak Sharif Ali

**Affiliations:** ^1^Department of Microbiology and Laboratory Sciences, SIMAD University, Mogadishu, 252, Somalia; ^2^Faculty of Medical Laboratory Sciences, Hematology Department, Al-Neelain University, Khartoum, 249, Sudan; ^3^Department of Hematology, Faculty of Medicine and Health Sciences, SIMAD University, Mogadishu, 252, Somalia

**Keywords:** anemia, Mogadishu, prevalence, SIMAD University, Somalia, students

## Abstract

**Background:**

Anemia remains a significant public health issue, affecting populations worldwide, particularly in low-income countries. Despite its widespread prevalence, no comprehensive studies have been conducted to assess the prevalence of anemia or its associated factors among university students in Somalia. This study aimed to assess the prevalence of anemia and its associated factors among undergraduate students at SIMAD University, Mogadishu, Somalia.

**Methodology:**

A cross-sectional study was conducted involving 264 participants from various faculties. Data on sociodemographic factors, medical and lifestyle factors, and hemoglobin (Hb) concentrations were collected. Statistical analysis including descriptive statistics, Pearson chi-square tests, and logistic regression was performed to determine associations between variables and anemia prevalence.

**Results:**

The mean age of participants was 20.3 ± 2.5 years, with an equal distribution of male and female students. The overall prevalence of anemia was 48.1%. Higher rates of anemia were observed among females (*p* < 0.001) and those not engaging in regular exercise (*p* = 0.001). Logistic regression showed that being male (OR = 0.409, 95% CI: 0.249–0.671) and engaging in exercise (OR = 0.299, 95% CI: 0.168–0.532) were protective against anemia. History of hospitalization showed a nonsignificant association with increased anemia risk (OR = 1.523, *p* = 0.121). A knowledge assessment revealed that 64.4% of the participants had good knowledge of anemia.

**Conclusion:**

Anemia was highly prevalent (48.1%) among undergraduate students, particularly among females and those not engaging in regular exercise. Male gender and physical activity were protective factors. Despite good knowledge levels, the burden remains significant. Targeted awareness campaigns, routine screening, and interventions promoting healthy lifestyles are essential to reduce anemia and its impact on students' health and academic performance. Further research should guide context-specific policy development.

## 1. Introduction

Anemia is a condition in which the number of red blood cells or their oxygen-carrying capacity is insufficient to meet physiological needs. Red blood cells contain a protein called hemoglobin (Hb), which is rich in iron and is essential for moving oxygen from the lungs to the other tissues. Thus, a decrease in this iron protein's levels impairs the body's ability to provide tissues and organs with oxygen, resulting in symptoms including shortness of breath, dizziness, headaches, and fatigue [1]. The etiology of anemia is complex and can include various factors such as poor nutrition (iron, folic acid, and vitamin B12), genetics (thalassemia and sickle cell), environmental pollution (lead), infectious diseases (malaria), socioeconomic status (low maternal education and low household income), age and gender, and autoimmune conditions (hemolytic anemia) [2]. Anemia has a major adverse effect on adult productivity and economic growth. According to a study that examined the costs associated with anemia in 10 developing nations, the median annual loss of physical productivity caused by anemia was estimated to be US$2.32 per person, or 0.57% of GDP. When the impact of anemia on cognition was considered, the total economic loss was calculated to be US$16.78 per person, or 4.05% of GDP [3].

Globally, over 1.93 billion people suffer from anemia, with over 89% living in developing nations [4]. This was a minor to moderately significant public health issue among undergraduates in countries with lower incomes. The prevalence among university students varies greatly, with 34% affected in Yemen, 55.3% in Bangladesh, and 67.35% among Saudi Arabian female students, respectively [2, 5, 6]. These figures show a worrying burden, especially for female students, which is probably caused by menstrual blood loss and nutritional deficiencies.

Anemia is a major public health concern in Somalia, affecting a sizable proportion of the population, particularly women of reproductive age and young children. According to data from a national survey done in 2009, anemia is a serious public health issue, with iron deficiency (ID) being especially common. According to the World Health Organization (WHO) [7]. Recent research indicates that the incidence of anemia in Somalia is dangerously high, with a pooled prevalence of 39.7% (95% CI: 26.3–53.1; I2 = 99.26%, *p* < 0.001) among 13 relevant studies comprising 3988 participants. This high frequency reflects the significant public health burden anemia imposes in the country [8].

Although anemia has a significant impact, there is a lack of research explicitly focused on anemia among university students in Somalia. This lack of focused investigation restricts our understanding of the frequency rates, underlying causes, and health consequences of anemia among students. It is essential to comprehend the frequency and risk factors of anemia in university students because they are particularly susceptible to this condition, and it has the potential to negatively affect their academic performance and overall health.

Therefore, the aim of the study was to fill this gap and to assess the prevalence of anemia and its associated factors among undergraduate students at Somali Institute of Management and Administration Development (SIMAD) University in Mogadishu, Somalia. This research seeks to contribute to local health policies and educational strategies by providing insights that can enhance student well-being and educational outcomes in similar settings worldwide.

## 2. Materials and Methods

### 2.1. Study Design and Time

From October 2023 to July 2024, an intuitional-based cross-sectional study was conducted in SIMAD University, Mogadishu, Somalia.

### 2.2. Study Area and Population

The target population of this study was undergraduate students at SIMAD, which is a private university in Mogadishu, Somalia, that was established in 1999. SIMAD began as a training center offering short-term courses and diploma programs in management, administration, and computer science. In 2011, the Board of Trustees approved the transformation of SIMAD into a full university. Over the years, it has built a strong reputation for offering exceptional higher education in Somalia.

### 2.3. Inclusion Criteria

Undergraduate students from different SIMAD University faculties who were interested in being part of our study were included in our research project.

### 2.4. Exclusion Criteria

We excluded students who were unwilling to participate, who were not enrolled as undergraduates at SIMAD University, or who were unavailable during the data collection days in our research.

### 2.5. Sampling Technique

A quota sampling technique was used to identify the different faculties. A convenience sampling was performed to select the individuals in each faculty. Quotas were set based on the number of students enrolled in each faculty to ensure proportional representation. Convenience sampling was used to select participants from each faculty based on logistical feasibility and participant availability during the study.

The total sample size was determined using single population proportion formula, resulting in 264 participants. Quotas were assigned proportionally to the student population in each faculty.

### 2.6. Selected Individuals in Each Faculty

Selected individuals in each faculty are presented in Table 1.

### 2.7. Sample Size Determination

The sample size was calculated by single population proportion formula considering proportion (*p*) of 20.6% prevalence of anemia based on study conducted in Ethiopia [4], Za/2 of 1.96, 5% of absolute precision, and 5% nonresponse rate. Hence, the total sample size was 264.

The sample size formula:

(1) $$\begin{array}{c} n={\left(\frac{Z\alpha }{2}\right)}^{2}\frac{P\left(1-P\right)}{E^{2}},\\ N=\frac{{\left(1.96\right)}^{2}*\left(0.206\right)*\left(1-0.206\right)}{{\left(0.05\right)}^{2}}=251. \end{array}$$

### 2.8. Data Collection Techniques

A questionnaire was used as the data collecting method. The questionnaire for this study divided into four main sections: sociodemographic factors, medical and lifestyle factors, nutritional status of the students, and general knowledge of the participants about anemia. Then we collected blood.

### 2.9. Laboratory Investigation and Sample Collection

Blood from finger pricks was collected in the microcuvettes using safety lancets. The fingers were cleaned with alcohol swabs before being pricked. Using cotton wool, the first drop of blood was removed, and a microcuvette was used to collect the second. The calibrated mission plus Hb meter was loaded with the blood sample in the microcuvette, and then Hb concentration was measured.

### 2.10. Variables of Interest

#### 2.10.1. Outcome (Dependent) Variable

• Prevalence of anemia, defined by Hb levels.

#### 2.10.2. Predictor (Independent) Variables

Demographic factors include age and gender.

Medical and lifestyle factors: body mass index (BMI), history of chronic diseases (diabetes and asthma), dietary habits, and sleeping duration.

### 2.11. Data Analysis

The data were entered into Excel 2016, edited, and cleaned for inconsistencies, missing values, and outliers and then exported to the SPSS 25.0 version for analysis. We used descriptive statistics to look at general patterns in sociodemographic, medical, and lifestyle factors, as well as knowledge and prevalence of anemia. To check participants' knowledge about anemia, we created a short questionnaire with six questions and asked them to answer it. The questions had multiple answers and every successful mention of that option resulted in 1 positive mark while failing to answer resulted in zero from that question. To evaluate the significant association of sociodemographic, medical, and lifestyle characteristics of the study participants with the incidence of anemia, statistical analysis of Pearson chi-square testing was performed and a *p* value < 0.05 was considered as significant result. Furthermore, to evaluate the odds ratio of occurrence of anemia, bivariate logistic regression analysis was performed on all the significant (*p* value < 0.05) from the Pearson chi-square test. Odds ratio greater than 1 was considered as significantly associated with anemia.

### 2.12. Ethical Considerations

Ethical clearance was gained from the ethical review thesis committee and the Faculty of Medicine and Health Sciences, SIMAD University. In addition, the purpose of the research was clarified, and the participants were informed of their rights to withdraw from the study. The ethics approval number for this study is 2025/019.

## 3. Results

A total of 264 blood samples were tested for the presence of anemia. A cross-tabulation analysis was conducted to clarify the correlation between anemia and other variables.

### 3.1. Sociodemographic Characteristics Among Undergraduate SIMAD University Students

Table 1 shows the responses of 264 students from nine faculties at SIMAD University, given in percentages. The participants had a mean age of 20.3 years with a standard deviation of 2.5 years. The youngest participant in the study was of 16 years with the oldest aged 39 years. Descriptive analysis of the age distribution revealed that 59.84% of the participants belonged to age group 19 years to 21 years, followed by 19.31% from 22 years to 24 years, and 17.5% aged 16 years to 18 years. Correspondingly, 2.27% of the participants were aged 25 to 27 years while 1.13% were aged more than 28 years. The study respondents equally comprised of male and female population, 132 participants from each gender. According to marital status, 97.34% of the participants were single in our study while only 2.66% were married. The anthropometric measurements of respondents showed that the respondents had a mean weight of 60 ± 10.93 kg in the study. 35.98% had a weight less than 55 kg while 51.51% weighed between 55 and 70 kg. Only 1.89% of the participants weighed more than 86 kg. The respondents had a mean height of 1.68 ± 0.9 m. The largest proportion of the participants (49.24%) had a height between 1.61 and 1.75 m. About 24.62% of the participants were 1.47–1.60 m tall, and 26.13% were 1.76–1.90 m tall. Also, the respondents had an average BMI of 21.48 ± 4.01, which means they were in the healthy range, since normal range of BMI is between 18 and 24.9. 48.1% of the participants had a BMI of 18.1–22.5 while 24.62 had 22.51 to 27. 18.56% had a BMI between 13.76 and 18 as well. Contrarily, 2.27% of the participants had a BMI between 31.51 and 36 (Table 2).

### 3.2. Medical Variables and Lifestyle Habits Among Undergraduate SIMAD University Students

Table 3 provides a complete overview of the different medical variables and lifestyle habits observed among the individuals. Malaria was the most widespread condition, accounting for 81.43% of cases, followed by parasite infections at a rate of 45.07%. In contrast, chronic disorders were less common, with a rate of 4.54%, while blood transfusions had a rate of 5.68%. Exercise had a high level of popularity, with 70.83% of people engaging in it. Specifically, gyms were the preferred location for exercise for 21.21% of people, while football accounted for 15.90% of exercise activities. The participants exhibited a wide range of food habits, with a significant proportion consuming red meat (88.25%) and fruits/vegetables (92.42%). Similarly, their sleep patterns varied, with the majority sleeping for 6–8 h per night.

### 3.3. Hb Concentration Among Undergraduate Students

The participants had a mean hemoglobin concentration of 11.25 ± 1. 87 g/dL. The prevalence of anemia, based on hemoglobin concentration, was 48.1% among the study participants.

### 3.4. Prevalence of Anemia in Different Faculties at SIMAD University

Our study revealed that the highest proportion of respondents (80.64%) from FMHS were anemic based on their Hb concentration. It was followed by FECO (64.28%), FMS (61.4%), FENG (46.150%), and FASS (40.9%). Respondents from FOC had the lowest prevalence of anemia at 13.23%.

### 3.5. Association of Sociodemographic, Medical, and Lifestyle Characteristics of the Students

Table 4 provides significant association of sociodemographic, medical, and lifestyle characteristics of the study participants with the incidence of anemia, and statistical analysis of Pearson chi-square testing was performed and a *p* value < 0.05 was considered as significant result. Upon analysis, gender and exercise were found to be significantly associated with the incidence of anemia.

### 3.6. Logistic Regression Analysis Among Students

Table 5 displays the results of a logistic regression analysis examining potential risk factors. It was found that engaging in exercise reduced the incidence of anemia by a factor of 0.29, with a *p* value indicating a statistically significant result. Gender was found to be a significant factor, showing that males had a decreased risk of anemia compared to females (0.409). Conversely, a previous record of being hospitalized was linked to a 1.52-fold higher occurrence of anemia, although this finding did not reach statistical significance.

### 3.7. Knowledge of Anemia Among Undergraduate Students

Table 6 displays the overall knowledge level, with a knowledge level of ≤ 8 defined as poor knowledge and a knowledge level of > 8 considered as good knowledge. 35.6% of the participants had poor knowledge of anemia while 64.4% had good knowledge about anemia.

## 4. Discussion

**Figure 1:**
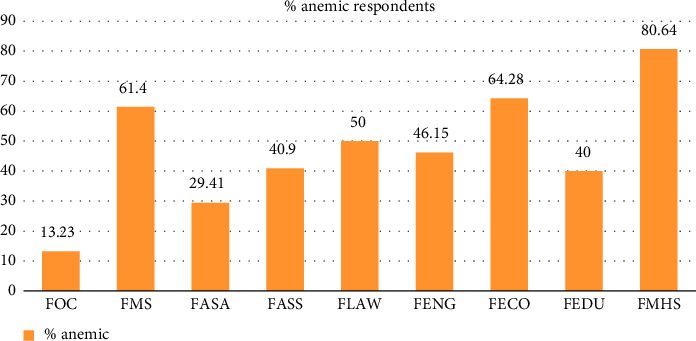
Prevalence of anemia in different faculties at SIMAD University.

The purpose of this study was to look at the prevalence of anemia among SIMAD University students and find risk factors. There were 264 participants in our study with an equal distribution of genders and a majority age range of 19–21 (59.84%). The majority of participants (97.34%) were single, and their average BMI was 21.48, their mean height was 1.68 m, and their mean weight was 60 kg (Table 2). Based on Hb levels, about half of the participants were anemic, with FMHS having the highest frequency (80.64%) (Table 7 and Figure 1). This high prevalence of anemia in FMHS (80.64%) is a noteworthy finding. One possible explanation is that FMHS has a higher number of female students, who are genetically predisposed to anemia due to variables such as menstrual blood loss and increased iron requirements throughout reproductive years. Medical students may experience higher levels of academic stress, irregular eating patterns, and inadequate sleep, all of which can increase their risk of anemia. FOC students, on the other hand, had the lowest prevalence (13%), possibly due to different lifestyle factors. These findings suggest that both biological and environmental factors contribute to anemia disparities among faculty.

Gender revealed a strong correlation, with females being more at risk, underscoring the need for gender-specific healthcare. Unexpectedly, it was discovered that malaria offered protection maybe because of immunological responses. Exercise was found to be protective, indicating broad health advantages. Anemia risk increased higher in hospitalization history, indicating the need for more research. Food choices did not directly correlate with the prevalence of anemia. Knowledge levels were not significantly associated with the occurrence of anemia, even though 64.4% of individuals had strong general knowledge regarding the condition (*p* = 0.752).

Anemia has negative effects on health and is a global public health concern that affects both developed and developing countries. 1.62 billion people worldwide have suffered, with developing country populations predicted to be affected at a rate of 36% [9]. As no previous studies about anemia had been conducted in the country regarding university students, the aim of this study was to find out how prevalent anemia was among SIMAD University students and what factors were linked to it. Based on criteria for Hb concentration, the results showed that about half of the individuals (48.1%) suffered from anemia. Zeleke et al. reported that the overall prevalence of anemia was 21.1% in their study [10]. According to Tilmilsina et al., 37.8% of undergraduate students were anemic [11], which are lower compared to our study findings. Lower prevalence was also reported (20.6%) among university students by Ayele et al. [4]. The prevalence in our study is comparable to that reported at Nobel Medical College in Nepal (53.7%), but when comparing medical students (FMHS), the frequency in our study (80.64%) is significantly greater [12]. This significant variation may stem from different socioeconomic levels, food habits, and healthcare access. Furthermore, Somalia's persistent political instability and economic issues may increase malnutrition and poor health outcomes, adding to the high prevalence of anemia.

In our study, total anemic people were 127 (49 males and 78 females), while total nonanemic people were 137 (83 males and 54 females). Among males, 49 out of 132 individuals (37.1%) are anemic. Among females, 78 out of 132 individuals (59.1%) are anemic. This agrees with a previous report from the WHO; the prevalence of anemia among females of reproductive age was found to be over 50%, and Somalia has been categorized as one of the countries experiencing severe levels of anemia [13]. Study conducted in Yemen revealed that 54% of anemic students were female while 46% of anemic students were male which is similar to our study in gender differences [2]. The prevalence of anemia is generally higher in females than in males, as reflected in the above findings, primarily due to biological factors [6, 5]. Females of reproductive age experience monthly menstrual blood loss and increased iron requirements during pregnancy and lactation to support fetal growth and breastfeeding. These physiological processes can make females more susceptible to ID and anemia. On the other hand, a study conducted in Nepal reported a higher prevalence of anemia among male students (44.7%) compared to female students (32.3%) [11].

In this research, findings indicate that students who participate in physical activity have a lower likelihood (odds ratio of 0.299) of being anemic compared to those who do not engage in exercise (Table 5). This suggests that exercise may act as a protective factor against anemia. Exercise has been shown to increase both total Hb and red cell mass, resulting in an improvement in oxygen-carrying capacity. The primary mechanisms behind this enhancement are thought to originate from the bone marrow, including increased erythropoiesis due to hematopoietic hyperplasia and improved hematopoietic microenvironment stimulated by exercise training. Additionally, hormone and cytokine-accelerated erythropoiesis also contribute to this improvement [14]. Although BMI was measured, its relationship to anemia was not significant in our study. However, a cross-sectional study conducted in 2003 indicated a higher frequency of ID among overweight and obese children and adolescents [15]. Another study from the National Health and Nutrition Examination Survey (NHANES III) confirmed that American children with overweight were twice as likely to be iron deficient as children with normal weight [16]. Similarly, according to Alam et al., serum iron and transferrin saturation were significantly lower in obese persons than in those with normal body weight [17]. As we can see, fat mass was discovered to be a substantial negative predictor of serum iron content, although the cause of this occurrence is unknown [18].

Present study reported that being hospitalized is associated with 1.523 times higher odds of being anemic. This finding aligns with research conducted in Italy by Migone De Amicis et al. [19]. They said that anemia is independently linked to longer hospital stays.

The study's limitation is from its cross-sectional methodology, which prevented the establishment of causal links. The study was conducted solely within a single university setting, limiting the ability to generalize the results. Also, the limited sample size may have compromised the capacity to apply the findings to a broader population.

## 5. Conclusion

This research investigated the frequency of anemia among SIMAD University undergraduate students, and it provided important light on the state of health. Our findings highlight the considerable burden of anemia, affecting nearly half (48.1%) of the students based on Hb concentration criteria. We conducted a thorough examination of 264 participants across various faculties. To prevent the prevalence of anemia among students, community health programs should encourage balanced diets high in fruits, vegetables, and iron. Also, community members should encourage regular physical activity programs because regular exercise dramatically lowers risk of anemia.

## Figures and Tables

**Table 1:** Selected individuals in each faculty.

Faculty	Participants
Faculty of Medicine and Health Sciences	31 participants
Faculty of Education	5 participants
Faculty of Economics	28 participants
Faculty of Management Science	57 participants
Faculty of Social Science	22 participants
Faculty of Engineering	13 participants
Faculty of Law	6 participants
Faculty of Computing	68 participants
Faculty of Accounting	34 participants
Total	264 participants

**Table 2:** Sociodemographic characteristics among undergraduate SIMAD University students.

Variables	Frequency	Percentage
*Age (years)*		
16–18	46	17.5
19–21	158	59.84
22–24	51	19.31
25–27	6	2.27
> 28	3	1.13
Total	264	100

*Gender*		
Male	132	50
Female	132	50
Total	264	100

*Marital status*		
Single	257	97.34
Married	7	2.66
Total	264	100

*Weight (kg)*		
40–55	95	36
56–70	136	51.51
71–85	28	10.60
> 86	5	1.89
Total	264	100

*Height (m)*		
1.47–1.6	65	24.62
1.61–1.75	130	49.24
1.76–1.9	69	26.14
Total	264	100

*BMI range*		
13.76–18	49	18.56
18.1–22.5	127	48.1
22.51–27	65	24.62
27.1–31.5	17	6.43
31.51–36	6	2.27
Total	264	

**Table 3:** Medical variables and lifestyle habits among undergraduate SIMAD University students.

Factor	Variable	*n* (%)
History of chronic diseases	Yes	12 (4.54)
	No	252 (95.46)

Any surgical procedures	Yes	33 (12.5)
	No	231 (87.5)

Ever received a blood transfusion	Yes	15 (5.68)
	No	249 (94.32)

Ever been hospitalized	Yes	108 (40.9)
	No	156 (59.1)

Ever had a parasite infection	Yes	119 (45.07)
	No	145 (54.93)

Malaria	Yes	215 (81.43)
	No	49 (18.57)

Use of any drug	Yes	7 (2.65)
	No	257 (97.35)

Heavy menstrual bleeding	Yes	46 (34.84)
	No	86 (65.16)

Exercise	Yes	187 (70.83)
	No	77 (29.17)

Type of exercise	Gym	56 (21.21)
	Football	42 (15.90)
	Jumping	7 (2.65)
	Running	11 (4.16)
	Pushup	9 (3.4)
	Yoga	6 (2.27)
	Walking	23 (8.710)
	More than one exercise	33 (12.5)

Sleep duration	3H	4 (1.51)
	4H	11 (4.16)
	5H	43 (16.28)
	6H	73 (27.65)
	7H	74 (28.03)
	8H	58 (21.96)
	10H	1 (0.37)

Meals per day	< 2 times	71 (26.89)
	2 times	105 (39.77)
	3 times	88 (33.33)

Red meat	Yes	233 (88.25)
	No	31 (11.75)

Fruit and vegetable	Yes	244 (92.42)
	No	20 (7.58)

Eating eggs	Yes	220 (83.33)
	No	46 (16.67)

Consumption of liver and kidney	Yes	204 (77.27)
	No	60 (22.73)

**Table 4:** Association of sociodemographic, medical, and lifestyle characteristics of the students.

Variable	Variable level	%	*p* value
Gender^∗^	Male	50	< 0.001
	Female	50	

Marital status	Single	97.34	0.628
	Married	2.66	

Chronic disease	Yes	4.1	0.746
	No	95.9	

Surgery	Yes	13.9	0.514
	No	86.1	

Blood transfusion	Yes	3.3	0.118
	No	96.7	

Hospitalized	Yes	45.1	0.2
	No	54.9	

Malaria	Yes	76	0.27
	No	24	

Parasite	Yes	45.9	0.803
	No	54.1	

Drug	Yes	1.6	0.343
	No	98.4	

Exercise^∗^	Yes	57.4	0.001
	No	42.6	

Red meat	Yes	84.4	0.073
	No	15.6	

Vegetable and fruit	Yes	90.2	0.198
	No	9.8	

Eggs	Yes	82	0.581
	No	18	

Liver and kidney	Yes	75.4	0.503
	No	24.6	

^∗^ *p* value less than 0.05.

**Table 5:** Logistic regression analysis among students.

Variable	Odds ratio	CI for odds ratio		*p* value
		Lower	Higher	
Gender^∗^	0.409	0.249	0.671	< 0.001
Exercise^∗^	0.299	0.168	0.532	0.001
Blood transfusion	0.617	0.184	2.073	0.435
Hospitalized	1.523	0.895	2.591	0.121
Vegetable and fruit	0.489	0.183	1.304	0.153

^∗^ *p* value less than 0.05.

**Table 6:** Knowledge of anemia among undergraduate students.

Variable	Score limit	*N*	%	*p* value
Poor	≤ 8	94	35.6	0.752
Good	> 8	170	64.4	

**Table 7:** Hemoglobin concentration among undergraduate students.

Hb conc (g/dL)	*N*	%
≤ 11	127	48.1
> 11	137	51.89

## Data Availability

Any reasonable request can be made to the associated author to acquire the processed data.

## Nomenclature

GDP: Gross domestic product
WHO: World Health Organization
Hb: Hemoglobin
RBC: Red blood cell
BMI: Body mass index
FMHS: Faculty of Medicine and Health Sciences
FEDU: Faculty of Education
FMS: Faculty of Management Science
FOC: Faculty of Computing
FASS: Faculty of Social Science
FASA: Faculty of Accounting
FLAW: Faculty of Law
FENG: Faculty of Engineering
FECO: Faculty of Economics
